# Head Motion and Inattention/Hyperactivity Share Common Genetic Influences: Implications for fMRI Studies of ADHD

**DOI:** 10.1371/journal.pone.0146271

**Published:** 2016-01-08

**Authors:** Baptiste Couvy-Duchesne, Jane L. Ebejer, Nathan A. Gillespie, David L. Duffy, Ian B. Hickie, Paul M. Thompson, Nicholas G. Martin, Greig I. de Zubicaray, Katie L. McMahon, Sarah E. Medland, Margaret J. Wright

**Affiliations:** 1 QIMR Berghofer Medical Research Institute, Brisbane, Australia; 2 School of Psychology, University of Queensland, Brisbane, Australia; 3 Centre for Advanced Imaging, University of Queensland, Brisbane, Australia; 4 Queensland Brain Institute, University of Queensland, Brisbane, Australia; 5 Virginia Institute for Psychiatric and Behavioral Genetics, Richmond, Virginia, United States of America; 6 Brain & Mind Research Institute, University of Sydney, Sydney, Australia; 7 Imaging Genetics Center, Keck School of Medicine, University of Southern California, Marina del Rey, California, United States of America; 8 Institute of Health Biomedical Innovation, Queensland Institute of Technology, Brisbane, Australia; Institute of Psychology, Chinese Academy of Sciences, CHINA

## Abstract

Head motion (HM) is a well known confound in analyses of functional MRI (fMRI) data. Neuroimaging researchers therefore typically treat HM as a nuisance covariate in their analyses. Even so, it is possible that HM shares a common genetic influence with the trait of interest. Here we investigate the extent to which this relationship is due to shared genetic factors, using HM extracted from resting-state fMRI and maternal and self report measures of Inattention and Hyperactivity-Impulsivity from the Strengths and Weaknesses of ADHD Symptoms and Normal Behaviour (SWAN) scales. Our sample consisted of healthy young adult twins (N = 627 (63% females) including 95 MZ and 144 DZ twin pairs, mean age 22, who had mother-reported SWAN; N = 725 (58% females) including 101 MZ and 156 DZ pairs, mean age 25, with self reported SWAN). This design enabled us to distinguish genetic from environmental factors in the association between head movement and ADHD scales. HM was moderately correlated with maternal reports of Inattention (r = 0.17, p-value = 7.4E-5) and Hyperactivity-Impulsivity (r = 0.16, p-value = 2.9E-4), and these associations were mainly due to pleiotropic genetic factors with genetic correlations [95% CIs] of r_g_ = 0.24 [0.02, 0.43] and r_g_ = 0.23 [0.07, 0.39]. Correlations between self-reports and HM were not significant, due largely to increased measurement error. These results indicate that treating HM as a nuisance covariate in neuroimaging studies of ADHD will likely reduce power to detect between-group effects, as the implicit assumption of independence between HM and Inattention or Hyperactivity-Impulsivity is not warranted. The implications of this finding are problematic for fMRI studies of ADHD, as failing to apply HM correction is known to increase the likelihood of false positives. We discuss two ways to circumvent this problem: censoring the motion contaminated frames of the RS-fMRI scan or explicitly modeling the relationship between HM and Inattention or Hyperactivity-Impulsivity.

## Introduction

Head motion (HM) is a well known confound for functional and structural neuroimaging studies [1–9]. Movement during functional MRI (fMRI) is responsible for greater error in the measurement of the Blood Oxygen Level Dependent (BOLD) signal (also in white matter and cerebrospinal fluid), for up to 10 seconds after the movement occurs [10, 11]. Motion during functional imaging tends to create spurious local functional connectivity (FC) and to weaken long range FC [1–3, 12, 13]. Similarly, HM can positively bias Fractional Anisotropy (FA) and mean Diffusivity (MD) measures computed from Diffusion Tensor Imaging (DTI) images [7, 8].

In fMRI analyses, several strategies are used to control or account for the effect of HM. A common approach is to regress out HM in the image processing [14] but also in downstream analyses [3]; this is the ‘nuisance covariate’ approach [3, 11, 14–18]. Even so, the effect of HM on FC may be too complex and dynamic to be completely removed by regression [3, 6, 10–12, 15]. A more recent approach, based on censoring of the highly noisy images [2], outperforms the regression based methods [10] ***(but see [19])*** by reducing the number of false positive results. Importantly, both these methods assume that HM is independent of the trait of interest (e.g., case-control status, or a cognitive or personality measure); thus the HM contribution to the fMRI signal is considered to be solely noise. However, in groups of patients with diseases such as multiple sclerosis or stroke there is increased HM compared to healthy controls [20–22], although the evidence is somewhat contradictory for schizophrenia [23–26]. Recently, HM (in both fMRI and DTI) was shown to be greater in impulsive individuals, in line with the findings from externalising disorders such as Attention Deficit Hyperactivity Disorder (ADHD) and conduct disorders [27]. In many studies the frequency of exclusions for gross motion was greater in ADHD [28–30] and autism cases [31, 32] compared to controls. This suggests a possible relationship between HM and the trait of interest that is yet to be investigated rigorously.

Many of these relationships with HM need to be replicated but, if confirmed, image processing pipelines will need to take such relationships into account to avoid a significant power loss and inflation of the false positive rate, as well as possible misinterpretations of the genetics of traits examined. An association between HM and the trait of interest during fMRI can potentially contribute to false positive discoveries and reduced power to detect effects at many levels. First is the exclusion criterion: common practice is to discard scans exhibiting motion greater than the voxel size [20]. Under dependency, such exclusion would introduce a sampling bias, as the exclusion would depend on the trait of interest. Second, HM association with the trait of interest might also contribute to false positive (and negative) results when groups are compared. False positive findings can arise from spurious (often short range) functional connectivities (FC) in the group with excess motion. False negative results are caused by the increased BOLD and FC variance in the motion group, which reduce statistical power. Thirdly, HM bias can be introduced by the experimental design: the nature and difficulty of a task can, for example, influence the frequency and intensity of stimulus correlated motion [29, 33]. Finally, there might be combinations of case/control status and experimental conditions that interact to induce even more motion as recently reported [20, 26].

In the resting-state literature, some functional brain differences in ADHD are consistently reported, such as reduced connectivity in the Default Mode Network [34, 35] and altered connectivity with visual, ventral attention and frontoparietal networks [35, 36] or with the partially overlapping cognitive control network [34]. These may explain attentional lapses in ADHD, where mind wandering controlled by the DMN might interfere with sustained attention [34, 35]. However, many other findings have not been replicated, for example the reduced connectivity between DMN and putamen [34, 37], reduced regional homogeneity in the DMN [38], inferior frontal gyrus and dorsal caudate [34, 39], together with aberrant brain activation or network properties [34, 40, 41]. Investigating the relationship between HM and ADHD symptoms such as inattention or hyperactivity-impulsivity might indicate whether inconsistent findings from prior resting-state studies could reflect a potential confound.

Here, we chose to focus on the relationship of HM with ADHD, following a recent study showing an association between HM and impulsivity [27]. We used the twin design to investigate how much of the association between these traits is due to environmental or genetic factors. Impulsivity-Hyperactivity and Inattention were measured by self and maternal report using the SWAN (Strengths and Weaknesses of ADHD Symptoms and Normal Behaviour) questionnaire [42]. We used the Jenkinson et al., metric [43] to summarise the 6 motion time series and compute the level of motion of each participant. This provides a computationally fast and direct measurement of the mean displacement over all brain voxels [12, 43, 44].

## Material and Methods

Measures of ADHD were collected in the context of the Brisbane Longitudinal Twin Study **[45]** in which twins are assessed at average ages 12, 14, 16 and 18 on a range of measures. During the in-person visits or during follow up online assessment, the mothers of the twins completed the SWAN questionnaire (detailed below) with ~65% asked to rate the twins’ current ADHD symptoms, where the mean age of the twins was 22 (years) (see **S2 Fig and Appendix A in S1 File** for a detailed explanation of the various waves and protocols). As young adults (mean age 26, range 18–32 years) the twins also completed a self-report version of the SWAN **[46]**, where the majority were asked to provide ratings of any current ADHD symptoms (see **S3 Fig, Appendix A and Table A in S1 File** for a detailed explanation of the various waves and protocols). In addition, at age 21–28 many of the same twins were invited to participate in the Queensland Twin Imaging (QTIM) study **[47]**, involving structural **[48]** and functional MRI (both resting-state and during a working memory task **[44, 49–51]**) as well as DTI **[52, 53]**.

### Participants

**Fig 1:**
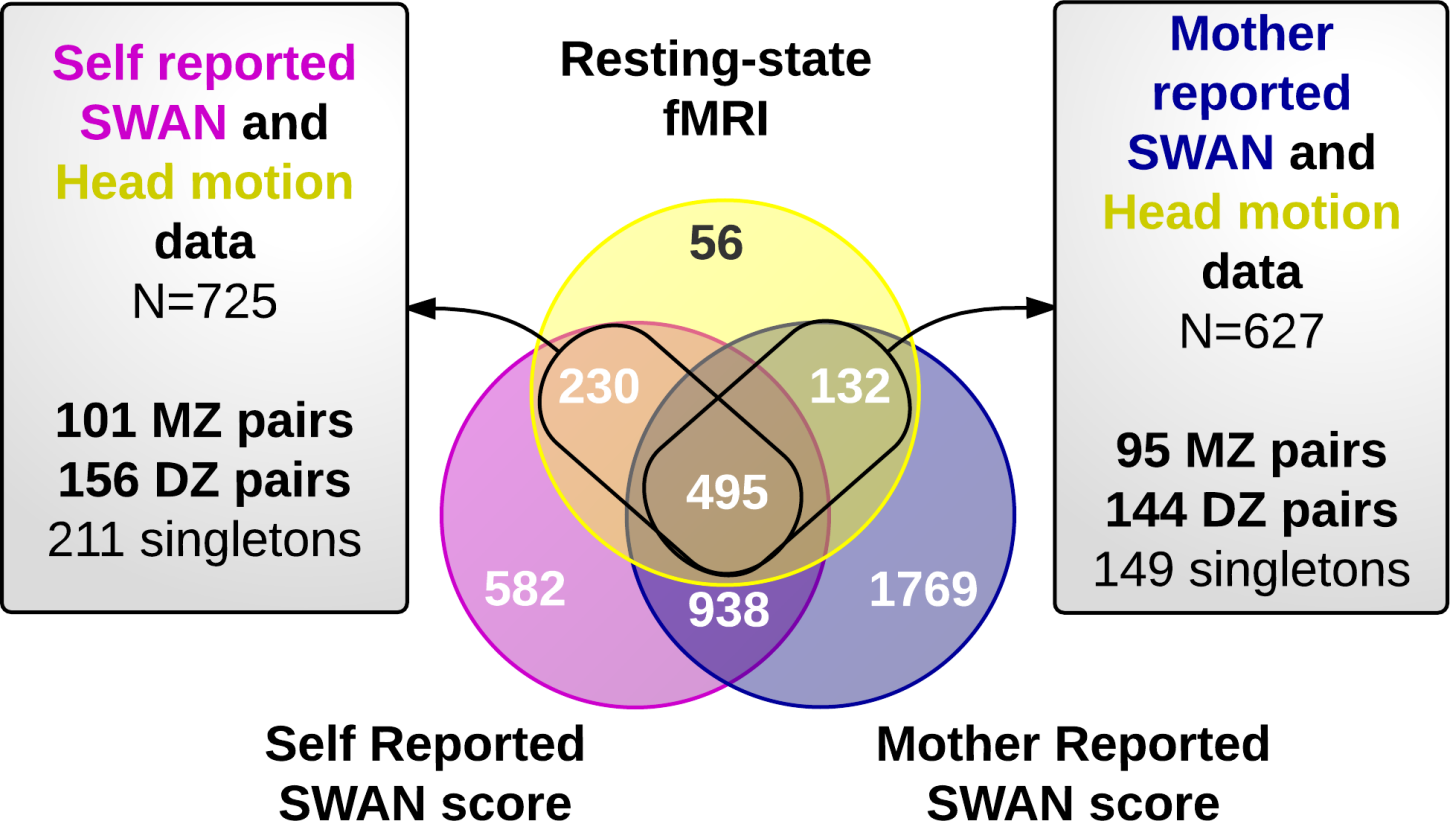
Overlap between SWAN scores and Resting-State functional MRI samples. This figure shows the sample size of the ADHD and QTIM studies. Individuals who exhibited gross motion during resting-state fMRI (N = 8), and siblings of a complete twin pair (n = 27) are not included. The sample used in the current study included those with both a SWAN score and HM measures, comprising two overlapping sub-samples (N = 725 and N = 627) as presented in the grey boxes. Number of monozygotic (MZ) and dizygotic (DZ) twin pairs (which include sib-pairs), and number of singletons are shown for each sub-sample.

We included all twins/siblings for whom head motion and at least one SWAN score were available (N = 892). For simplicity, we selected one pair of twins (or sibling pair) from each family resulting in the exclusion of 27 individuals. Eight additional participants were excluded for gross motion that occurred during resting-state fMRI (described in **Table A in S1 File**). The final sample included 857 individuals from two overlapping sub-samples (**Fig 1**). See [54] for a description of the resting state sample, [55] for the mother-reported SWAN sample and for a partial description of the self-reported sample (ongoing study). We also describe the currently available sample with self reported SWAN data in **Table A in S1 File**.

Fifty-seven (57) non-twin singletons remained in the final sample and provided 22 sibling pairs that were pooled with the dizygotic (DZ) twin pairs in the genetic analyses. Mother reported SWAN scores and HM were available for 627 participants (95 monozygotic (MZ) twin pairs, 144 DZ pairs, 149 singletons) and self reported SWAN scores and HM on 725 individuals (101 MZ pairs, 156 DZ pairs and 211 non-paired singletons). Our combined final sample included slightly more females (64%) with a mean age of 22 years for the resting-state scan (**Table A in S1 File and S1 Fig**). On average, there was 1.2 years (range 3 days-3.9 years) between the MRI scan and the SWAN rating from the mother, with 54% of the participants completing the scanning first. The interval between the MRI scan and collection of the self report SWAN scale was 3.1 years on average (range 6 days-7.1 years); 98% of participants were scanned first.

Zygosity was established objectively by typing nine independent microsatellite polymorphisms (PIC N 0.7) in the ProfilerPlus™ set using standard methods, and was later confirmed for 80% of the sample genotyped on the 610 K Illumina SNP chip[56]. Prior to imaging, participants were screened (by self-report) for being right-handed and for significant medical, psychiatric or neurological conditions, including head injuries, a current or past diagnosis of substance abuse, and for current use of medication likely to affect cognition.

Written informed consent was obtained from all participants including a parent or guardian for those aged less than 18 years. QIMR Human Research and Ethics Committee (HREC) approved SWAN data collection. Data collection for SWAN self report was also approved by the Virginia Commonwealth University Institutional Review Board (IRB). The QTIM study was approved by the ethics review boards of the Queensland Institute of Medical Research, the University of Queensland, and Uniting Health Care, Wesley Hospital, Brisbane. QTIM participants received an honorarium in appreciation of their time.

### Head motion Measurement

We extracted Head motion from a 5m 19s resting state fMRI recording [47]. Rigid body registration [57] was performed using SPM8 [58] and the DPARSFA toolbox [59], to realign the 150 images acquired during the scanning session. This processing step is unchanged in the new software version: SPM12 [60]. Three translational and three rotational parameters were calculated for each image to fully describe its position from the first, and reference, image. Using the standard exclusion criteria for 3-4T fMRI studies, we identified 8 outliers with translation (in one of the directions x,y or z) greater than 3mm, or rotation (around one of the axes) greater than 2 degrees. These individuals with gross motion were excluded from the analysis due to their possible large influence on the results. Characteristics of these individuals are reported in **Table A in S1 File**, where they are compared to the rest of the sample and **S1 Fig**.

For the final sample comprising 857 participants (see **Fig 1**) we summarised the 6 motion time series by computing the Jenkinson et al., metric of HM [43, 61] which offers the advantages of being comparable across studies (independent of centre of rotation), highly correlated with the metric of reference (average voxel-wise displacement) but is much faster to compute [12]. It can be calculated using the formula: $\sqrt{\frac{1}{5}{\text{R}}^{2}\text{trace}({\text{A}}^{\text{T}}\text{A})+{\left(\text{t}+\text{A}{\text{x}}_{\text{c}}\right)}^{\text{T}}(\text{t}+\text{A}{\text{x}}_{\text{c}})}$ where ${\text{T}}_{2}{{\text{T}}_{1}}^{-1}-\text{I}=\left[\begin{array}{cc} \text{A} & \text{t}\\ 0 & 0 \end{array}\right],\;{\text{x}}_{\text{c}}$ being the centre of the volume (software specific), T_1_ and T_2_ the transformation from volumes of interest (frames at time d and d+1) to reference volume (usually the first frame). A is therefore the 3x3 matrix of rotation and t the vector of translations of the affine transformation between volumes of interest. R is the arbitrarily chosen radius of the brain, here 80 mm [43]. Hence, the Jenkinson et al. metric aggregates translation and rotation movements that we showed to be genetically homogeneous [44]. We log-transformed the HM metric to enhance normality of the residuals in the linear models. One outlier, with a composite HM greater than 4sd from the mean (5.4 standard deviations from mean), was identified but included considering the minimal impact of a single observation on the result. The average level of motion observed in the sample, as well as the MZ and DZ twin pair correlations aligned with our previous report of heritability of HM [44] and is reported in **Table 1.**

**Table 1:** Levels of Inattention, Hyperactivity-Impulsivity and Head Motion in the final sample.

		Mother reported (N = 627)		Self reported (N = 725)		All (N = 857)	
		Inattention	Hyperactivity- Impulsivity	Inattention	Hyperactivity-Impulsivity	Head Motion (log-transformed)	Head Motion (mm)
		Mean (sd)	Mean (sd)	Mean (sd)	Mean (sd)	Mean (sd)	Mean (sd)
**All individuals**		-1.04 (1.14)	-1.06 (1.10)	-0.51 (0.59)	-0.50 (0.62)	-2.59 (0.35)	0.080 (0.032)
**Sex**	**F**	-1.24 (1.08)	-1.21 (1.07)	-0.57 (0.58)	-0.57 (0.60)	-2.62 (0.34)	0.078 (0.031)
	**M**	-0.70 (1.17)	-0.81 (1.10)	-0.40 (0.62)	-0.36 (0.64)	-2.53 (0.35)	0.085 (0.032)
**Wave**	**1st**	-1.04 (1.14)	-1.07 (1.09)	-0.64 (0.33)	-0.28 (0.55)	NA	NA
	**2nd**	-0.64 (0.94)	-0.70 (1.36)	-0.61 (0.66)	-0.62 (0.68)	NA	NA
	**3rd**	NA	NA	-0.48 (0.58)	-0.47 (0.60)	NA	NA
		Pair correlation	Pair correlation	Pair correlation	Pair correlation	Pair correlation	
	**MZ**	0.72	0.86	0.47	0.31	0.41	0.43
	**DZ**	0.15	0.51	-0.069	-0.046	0.16	0.15

The description of excluded participants for excessive motion (greater than voxel size) is available in **Appendix B and Figures D, E in S1 File** but the comparison with included participants is limited by the small number of excluded participants (N = 8,**Table B in S1 File**).

### ADHD measurement

Dimensions of ADHD (Inattention and Hyperactivity-Impulsivity) were assessed using 18 items from the SWAN questionnaire [42]. This includes 9 items for the Inattention dimension and 6+3 for Hyperactivity and Impulsivity. The items correspond to the symptoms defined in the DSM-5 (and DSM-IV) and each of them is rated on a Likert-type scale. Raters are instructed to use average individual (of a specified age) as a reference. Mothers were provided 7 rating possibilities for their children: far below average, below, slightly below, average, slightly above, above and far above average. For self rating a five-point Likert-type scale was used: far below average, below, average, above and far above average. In both cases, “average” was rated 0 and “far below average” was given the maximum score. For each dimension symptom ratings are averaged to create a continuous score, a higher score indicating higher levels of ADHD. This instrument has high reliability (r = 0.82) and internal consistency (α = 0.88), as well as good agreement with the Disruptive Behaviour Rating Scale [62] or ADHD diagnosis [63]. In addition, the SWAN scale may more precisely describe the individuals at the left end of the distribution (low Inattention and Hyperactivity-Impulsivity) [63–65], which better suits the study of population samples and estimation of twin correlations [64, 65].

In the previous version of the DSM (DSM-IV), ADHD was only defined for children and adolescents, but the new edition (DSM 5) raises the age when the symptoms can be documented, allowing diagnoses to be made on adults. Thus, the SWAN scores based on the DSM symptoms can be safely used in young adults and jointly analysed together with the observations at earlier age providing that age and sex are used in the analyses to remove their confounding effects [51, 66, 67]. Indeed, results from a large community-based sample in the US indicate persistent sex differences across all age groups, as well as a steady decrease of mean scores from childhood to adulthood [66].

Here, SWAN scores were, on average, greater for males than females, consistent with previous observations of the same data [55] and with independent analyses of impulsivity and inattention [51, 66, 67]. In addition, mean and variance differences in Inattention and Hyperactivity-Impulsivity between waves (see **Table 1**), led us to consider wave and design (prospective vs. retrospective) as covariates, in addition to sex and age. Previous research on this dataset showed similar variance decomposition in each of the waves or design samples, suggesting that after accounting for the effects of age and wave the maternaland self-report data may be jointly anlaysed [55]. Finally, a negative contrast effect has been previously found in ADHD scores [55, 68–74], whereby a high rating of one twin is associated with a lower rating of the co-twin. This contrast effect can arise from phenotypic interaction (the behaviour of the hyperactive twin might impact the co-twin’s behaviour, and vice versa) or more likely from a rater effect (the more hyperactive a twin is perceived, the less so is the co-twin perceived) [69]. Negative contrast is usually suggested by reduced twin pair correlations for some raters (e.g. parent report in ADHD), which can sometimes yield negative DZ correlations. Using the full twin sample (N = 3,223), a significant contrast effect was found for maternal report of Hyperactivity-Impulsivity and self reported Inattention [55]. Here, we tested the effect of the number of siblings in a family on SWAN scores, which could attenuate (up to a third) the rater effect as mother of large families tend to judge more accurately how their twins compare to average [72]. In our sample, families comprised 3 children on average (sd = 1.0, range = 2–7), which despite being the best measure available may be under-declared and dated.

### Statistical analyses

Analyses were performed in R 3.1.0 [75] using OpenMx [76], which allows the use of family data when estimating the correlation between HM and the SWAN scores, the effect of covariates (age, sex, wave, design, number of siblings, time difference between scoring and scanning), heritability (univariate variance component ACE/ADE modelling), as well as the estimation of genetic correlations (bivariate ACE or ADE models, with Cholesky decomposition). Additional packages were used to facilitate plots and data handling [77–79]. ACE or ADE models decompose the variance of a trait into an “additive genetic” (A) component that estimates the additive contribution of the genetic factors to individual differences; “shared environment” (C) that captures the effect of a common environment/household factor on a sib pair; “dominant genetic” (D) for the non-additive genetic contribution, and “unique environment” (E) that corresponds to the effect of person-specific environmental factors or measurement errors on the trait variance. When several traits are modeled together (bi- and multivariate analyses), sources of variances are further decomposed into sources common between several traits and specific to each trait. This methodology is well known and well described [80–82]; see [83, 84] for a user-friendly description. MZ and DZ twin pair correlations (Table 1) suggested the presence of genetic factors for all traits (rMZ>rDZ), with possible dominant genetic effects for the SWAN scores (rMZ>2rDZ). To estimate genetic and environmental correlations between the SWAN scores and HM, we used an AE decomposition of variance, to maximize power in our limited sample. In such model, the A factor would gather most of the D contribution to variance (when present) and genetic correlation can be interpreted as mostly additive or mostly dominant depending on the SWAN score considered. Correlations were corrected for covariates that reached significance in the univariate models, as well as time difference between scoring and scanning, which could mediate or confound the relationship between motion and ADHD levels. Significance of the correlations, covariates and model fit comparison were tested using likelihood ration test (nested models). We reported estimates, p-value and degrees of freedom of the test statistic law.

## Results

### Preliminary tests–covariate effects, homogeneity of sampling, and genetic effects

Overall, covariate effects were small or non-significant. For all SWAN scores males scored on average 0.18 to 0.56 points higher than females **(Δdf = 1, p-value < 4.7E-4).** Age at MRI had a small effect on HM with an average reduction of 0.6% in HM per year of age **(Δdf = 1,** p-value = 4.0E-4) (**Table C in S1 File**). HM for males was greater than for females **(Δdf = 1,** +3.2% on average, p-value = 1.4E-3). Significant covariates were regressed out in subsequent analyses.

Two tests suggested variance heterogeneity between zygosity groups (Table C in S1 File) but only the variance differences in HM between groups corresponded to a true difference: the variance differences in self reported Inattention being driven by one outlier (S6 Fig and Table D in S1 File).

Finally, test for “quantitative sex limitation” was significant for mother reported Hyperactivity- Impulsivity, suggesting different levels of heritability between sexes. Covariances for female pairs (0.97 in MZ, 0.74 in DZ) were higher compared with males (0.64 for MZ, 0.27 for DZ, Δdf = 2, p-value = 0.024, see Table C in S1 File) pointing to a higher shared environment effect in the female group. Presence of genetic factors (MZ covariance > DZ covariance) was significant for all variables tested (Δdf = 1, p-value<2.2E-4, Table C in S1 File). As a consequence, in estimating heritability we fitted a general sex limitation model for mother-reported hyperactivity-impulsivity (allowing the A, C/D and E components to be different between males and females) and standard “2-zygosity groups” ACE or ADE models for the 3 other SWAN scores. In HM univariate modelling, we allowed the variance to be different between female same sex and opposite sex pairs.

Heritability of Inattention was between 0 and 43% for maternal report with a dominant genetic effect explaining another 32 to 83% of the variance. For mother reported Hyperactivity-Impulsivity we found the additive sources to explain between 88 and 94% of the variance in females. The A component was only explaining 63 to 87% in males. The general sex limitation model outperformed standard ACE confirming tests from the hypothesis testing (supplementary Table 3). Genetic correlation (r_g_ = 1) between males and females suggested that genes influencing Hyperactivity-Impulsivity are the same, even if they explain different proportion of variance within sexes. Variance of self-reported inattention was also attributed to additive (0–22%) and dominant (16–60%) factors, while additive sources of variances explained less that 34% of the self rated Hyperactivity-Impulsivity variance. Heritability of HM was estimated to be h^2^_HM_ = 0.40 [0.26,0.53] (Supplementary Table 4 and Supplementary Appendix 3).

### Genetic and environmental correlations of HM and ADHD

The phenotypic correlation between HM and the SWAN scores was fairly homogeneous, with correlation coefficients **ranging from 0.09 to 0.20** (**Table 2**). All phenotypic correlations were significant, as suggested by the 95% CI (**Δdf = 1,** p-values: 7.4E-5, 2.9E-4, 0.017 and 2.0E-3).

Most of this correlation arose from common genetic factors, with the genetic correlation (0.19 to 0.40) being greater than the environmental correlation (0.03 to 0.12). Genetic correlation coefficients with HM were significantly different from 0 for maternal scoring (**Δdf = 1,** p-value = 3.0E-2 for Inattention, 3.9E-3 for Hyperactivity-Impulsivity). The correlation of HM with self report Inattention or Hyperactivity-Impulsivity did not reach significance as indicated by the CI (p-value = 0.29 and 0.074). Correction for age and age difference between ADHD scoring and MRI scanning did not significantly improve the fit of the models (Δdf = 4, p-value = 0.23 for mother reported inattention, p-value = 0.30 for Hyperactivity-Impulsivity, 0.30 for self scoring Inattention and 0.17 for Hypeactivity-Impulsivity), and left the correlation estimates unchanged.

**Table 2:** Phenotypic, genetic and environmental correlations [95% CI] between HM and the four SWAN scores.

	Maternal report scores		Self report scores	
	Inattention	Hyperactivity / Impulsivity	Inattention	Hyperactivity / Impulsivity
**Phenotypic correlations**				
Head Motion	0.17 [0.09,0.25] p-value = 7.4E-5	0.16 [0.07,0.24] p-value = 2.9E-4	0.09 [0.02,0.17] p-value = 0.017	0.12 [0.04,0.19] p-value = 2.0E-3
**Genetic correlations**				
Head Motion	0.24 [0.02, 0.43]	0.23 [0.07,0.39]	0.19 [-0.20,0.56]	0.40 [-0.05,1.00]
	p-value = 0.030	p-value = 3.9E-3	p-value = 0.30	p-value = 0.074
**Environmental correlations**				
Head Motion	0.12 [-0.07,0.31]	0.05 [-0.14,0.24]	0.07 [-0.10,0.25]	0.03 [-0.13,0.20]

Correlations were calculated using OpenMx [76] to take into account the relatedness in our sample when computing p-values or confidence intervals. Genetic correlations were estimated using a bivariate AE model, correcting for sex, age and age difference between scoring and scanning.

## Discussion

Here, we investigated the relationship between head movement during RS-fMRI and SWAN scores of ADHD in a young, non-clinical population sample. We found head motion to be significantly correlated with self and maternal reports of Inattention and Hyperactivity-Impulsivity. When breaking down this association into its genetic and environmental components, we detected pleiotropy (shared genetic effects) between HM and the SWAN sub-scales. Therefore, treating HM as a nuisance covariate in an RS-fMRI study of ADHD would likely remove some of the signal of interest.

Our findings align with the (phenotypic) association of HM with (self reported) impulsivity in a similar age group (r = 0.10) and in children (r = 0.34) [27]. We also found a significant phenotypic correlation between HM and inattention, which has not been reported before [27]. We expanded prior findings [27] by showing that the association was driven by genetic factors common to HM and ADHD rather than by experimental conditions (environmental). Indeed, the genetic correlation coefficients were around 0.2 between HM and the SWAN scores. Correlations were significant between HM and the maternal report scores However, they did not reach significance for the less reliable self-report scores. Environmental correlation estimates, on the other hand, were much lower and remained non-significant. This suggests that most of the phenotypic correlation between HM and Inattention or Hyperactivity-Impulsivity arises from pleiotropy between these traits. In other words, the association between in-scanner motion and Hyperactivity-Impulsivity or Inattention can be attributed to genetic factors shared between the traits.

**Fig 2:**
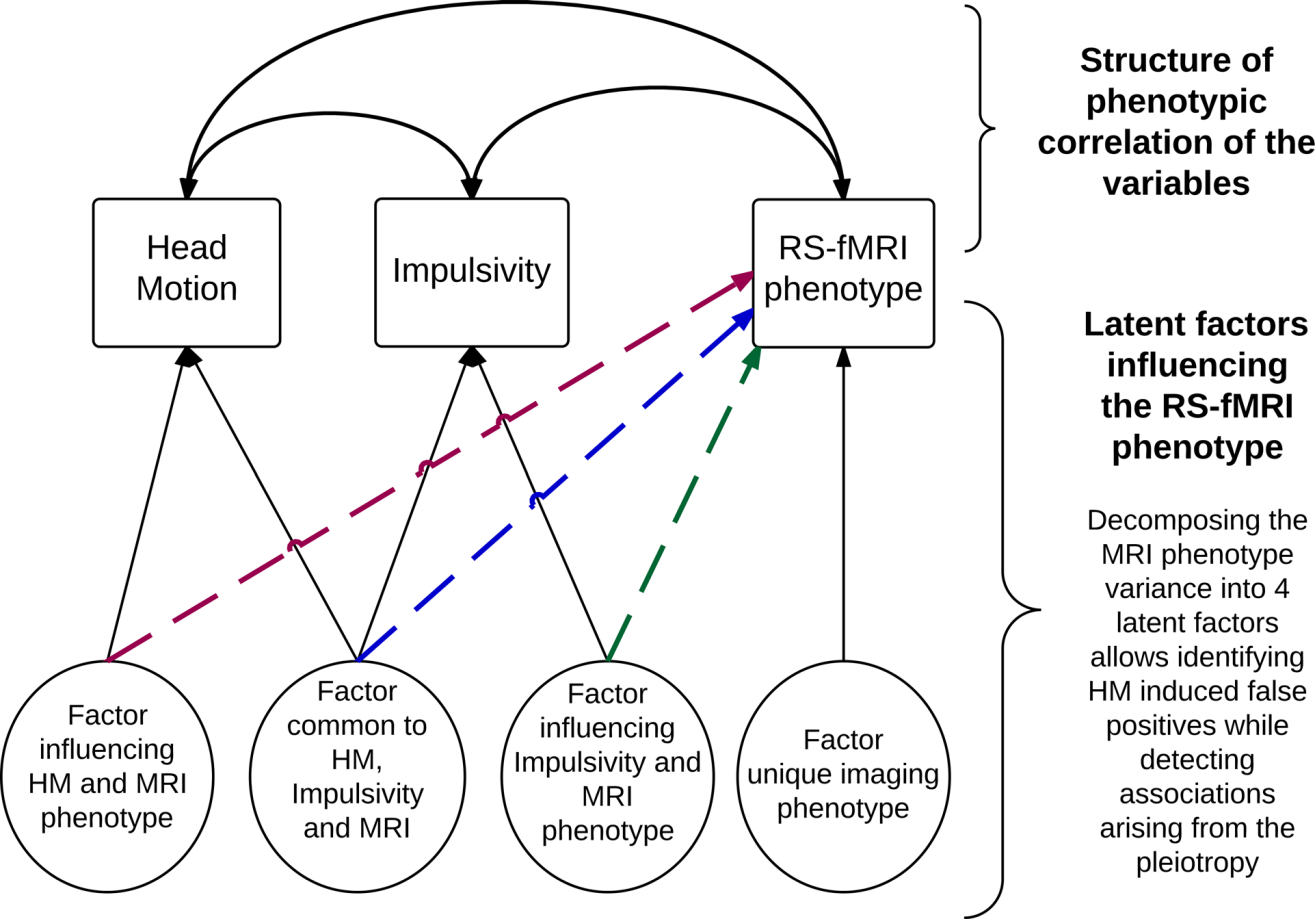
Structural Equation Model that can disentangle the effects of HM and psychological trait (here impulsivity) on RS-fMRI phenotype. The RS-fMRI phenotype variance is decomposed into 4 factors (or latent traits). The first one influences HM and the brain phenotype and captures false positive findings induced by HM. The second factor is common to HM, Impulsivity and the MRI phenotype. This source of variance can be regressed out by HM regression thus reducing power of detecting some brain changes associated with Impulsivity. The third factor influences Impulsivity and the RS-fMRI phenotype, it is conserved after HM regression. Finally the last latent factor is unique to the imaging phenotype and gathers the sources of variance not accounted by the 3 others. The use of twin and family data allows breaking down each of these factors into genetic and environmental components, thus showing light on the genetic structure of the associations.

The genetic nature of the association between HM and maternal reports of Hyperactivity-Impulsivity raises concerns about the way HM is handled during the image processing pipeline and to a lesser extent during downstream analyses of neuroimaging studies. Indeed, our results indicate HM appears to contain meaningful information for the study of hyperactivity-impulsivity. Regressing out HM when studying ADHD (or Inattention or Hyperactivity-Impulsivity) using neuroimaging would result in a loss of information in the analysis, with a direct increase of the false negative rate. For example, in an analysis of brain changes associated with ADHD, factors contributing to ADHD, brain phenotype and HM would suffer from diminished power. For example in **Fig 2**, HM regression would limit the detection of common factors (common to HM, brain phenotype and ADHD: in blue) that we estimated to represent about a quarter of the ADHD genetic factors. HM regression could also reduce heritability of processed brain phenotypes that are genetically correlated with HM. A similar criticism can be made about HM regression in downstream analyses that typically include HM as a covariate in across subjects analysis [3]). However, this may have little effect on the results as it is usually performed after HM regression in image processing, when most of the HM effects on the phenotype have already been removed. Thus, if it is accepted that head motion needs to be addressed to avoid false positives [1–6], we might have to consider approaches that do not rely on HM regression in rs-fMRI studies of ADHD. As a consequence, the RS-fMRI studies of ADHD [34, 40, 41, 85, 86] that performed HM regression might have suffered from reduced power, leading to some of the inconsistent results reported. Conversely, the recent study of neurobiological changes associated with HM [87] might have been partially confounded by trait Inattention or Hyperactivity-Impulsivity. Overall, our results could explain some of the network structure observed within the variance removed by HM regression [88]. Indeed, the ‘nuisance covariate’ approach would likely remove highly structured signal corresponding to brain networks of Inattention/Hyperactivity, as well as noise [88].

A similar limitation may apply to Global Signal Regression (GRS) whose independence of the trait/disease of interest has been debated. Factors influencing the global signal in RS-fMRI are only partially known: cardiac pulsation and breathing [89, 90], head motion [10], intrinsic spontaneous physiological low frequency oscillations [90], cerebro-spinal fluid [91]. In addition, their impact on the global signal might not be uniform across the brain [90, 91]. For example, cardiac pulsation is more prominent in the base of the brain, and respiration affects mostly prefrontal and occipital lobes [90]. As a result, GSR regresses out physiological noise [89] and increases specificity in positive functional connectivity [16], but changes the distribution of FC across the brain [89, 92] and may exacerbate motion distance-dependent bias [13], which could lead to false positives or even negative FC [89]. This suggests that, similar to HM, an assessment of the association between global signal and trait of interest is required to understand how GSR impacts the results. Preliminary results suggest that global signal could be heritable, with GSR reducing heritability estimates of graph metrics calculated from RS-fMRI images [54].

In addition, we presented only weak evidence that the exclusion of scans based on motion could cause a sampling bias (**Table A in S1 File**). We were limited by a very small sample (8 individuals with gross motion, and only 4 with self reported scores for Inattention and Hyperactivity/Impulsivity; **see Table A in S1 File**). However, if exclusions were found to be associated with levels of Inattention of Hyperactivity-Impulsivity, researchers would have to reconsider gross motion exclusion in rs-fMRI processing.

Some already available preprocessing methods might be employed to avoid gross motion exclusion and regression of HM (or other confounds), such as within-subject image volume censoring [2, 10]. This approach, however, does not guarantee comparability of HM between groups, as only the noisiest volumes are excluded [10]. Similarly, it might not completely prevent sampling bias as individuals with high numbers of noisy volumes are still recommended to be excluded [10]. Nevertheless, censoring could still be considered a valid approach, if it can be shown that both exclusion and remaining estimates of motion between frames are independent of the trait of interest (e.g. ADHD). If motion scrubbing were to prove insufficient in removing HM, or if GSR had to be avoided, structural equation modelling (SEM i.e. a multivariate approach) could be employed as an alternative to prevent researchers having to choose between increased false positives or power loss in RS-fMRI studies. For example, SEM could allow decomposing the variance of the MRI phenotype into motion specific, Hyperactivity-Impulsivity specific and that which is common to HM and Hyperactivity-Impulsivity. Therefore, one could identify brain changes specific to ADHD but also brain differences driven by the combination of HM and hyperactive-impulsive profile (see **Fig 2**for a path model with impulsivity as example). SEM more generally applies to all study RS-fMRI data in the presence of dependency between a confounding factor (HM, or global signal) and a trait of interest. It is also compatible with twin and family data for which the variance can be further decomposed into genetic and environmental factors [80]. Finally, it can accommodate phenotypes of high dimension; such as voxel-wise brain maps while modeling the local correlation of the voxels.

***Another alternative to motion regression or censoring could be regression of principal components of the voxel-wise nuisance signals from WM and CSF (aCompCor) [93]. Indeed, such correction appeared to make censoring redundant in a healthy adolescent sample, while also accounting for some of the global signal [19]. However, the interpretation of the principal components remains unclear and they may also be associated with the trait of interest, hence removing some meaningful signal.***

Finally, beyond considerations of RS-fMRI image processing, the genetic correlations measured here suggest that HM could be a valuable endophenotype [94] and a risk/trait biomarker [95] in RS-fMRI studies of Hyperactivity-Impulsivity, Inattention and, by extension, for ADHD. A similar trait biomarker for ADHD is activity levels (measured using accelerometers), which has also been genetically associated with ADHD [96, 97]. However, despite being two widely-used measures of movement, the extent to which activity level (large voluntary movement) and HM at rest (microscopic and involuntary) are caused by the same genes and environment factors is still unknown.

We showed some of the correlations to be reliable across scores (self or maternal report), despite observations acquired at different ages. Even so, the association between self report scores and HM was weaker, which could be explained by noisier measurement [55] and perhaps by the longer time difference between self report collection and scanning. However, in our sample, age and time difference between scoring and scanning were not associated with mean phenotypes and left the correlations unchanged. This might be due to observations made mostly at adult age when we can expect greater stability of personality and ADHD traits, as shown by higher SWAN scores test-retest coefficients [63]. Nevertheless, we cannot exclude that the effect of time difference may be non-linear and so undetected. Another limitation of our study was that we could not separate hyperactivity from impulsivity in the analysis, nor could we investigate subtypes of impulsivity that might not contribute to the HM increase [27]. In addition, RS-fMRI was acquired last in the ~50 minutes scanning session, which could potentially accentuate the strength of the relationship between HM and Impulsivity or Hyperactivity by increasing movement in the impulsive or hyperactive group. Finally, we have used a healthy population based sample and the correlation between HM and Inattention or Hyperactivity-Impulsivity (thus the influence of HM regression on the results) might be larger in a clinical sample where the contrasts between participants are increased, as suggested by correlations in a case-control sample [27]. Finally, sampling could also be a limitation as we only included imaged participants, who were screened for past diagnoses of mental illness and prescription medicine [47]. Hence, the sample used here might no be representative of the unselected cohorts studied for ADHD. However, heritability of SWAN scores (including higher contribution of environmental sources on the variance of the maternal report of hyperactivity/impulsivity) aligned with prior findings from full datasets [44, 55] and from independent cohorts that used the SWAN instrument [65, 73, 98, 99] (see Appendix C and Table D in S1 File). In addition, we observed higher SWAN scores and head motion in males compared to females, consistent with previous reports on the full cohorts [44, 55] and results from independent studies [3, 51, 66, 67], including meta-analysis on case control status [67], continuous performance test [51] for ADHD. As a consequence, we can be confident that the results reported here are unlikely to be due to sampling bias. Sex differences in ADHD have been attributed to lesser inhibitory control in males and higher internalizing of struggle in females [51]. On the other hand, very little is known about causes of higher motion in men but we can hypothesise that it might be partially influenced by the same factors, considering the association between the traits. Differences in heritability between maternal and self report scores are ubiquitous in ADHD [73, 100] and may be partially due to increased measurement error in self report, as suggested by the test-retest reliabilities that often sets the upper bound of the heritability estimates [101] (test-retest is around 0.45 for self report and 0.80 for mother report [55]). Lower reliability of psychiatric self-reports has been attributed to lower self-awareness, perspective taking, recall, reasoning ability, and expressive skills in adolescents/young adults [67]. However, there is evidence that, despite the differences in reliability, heritability of maternal report of hyperactivity might also well be overestimated as twins perceived as identical tend to be given more similar scores (expectancy effect) [70].

## Supporting Information

S1 Fig Age at resting-state functional MRI in the final sample (TIFF)

S2 Fig Age at mother report in the final sample, broken down by wave. Wave 1 corresponds to mother report acquired during the clinical visits of the twins at age 14. Wave 2 was collected using an online questionnaire several years after the clinical visits. If the twins were older than 20 years old when mothers were contacted, they were asked to rate current symptoms (prospective design). If twins were not 20, mothers were instructed to “think back to when he/she was in primary school” (retrospective design).
(TIFF)

S3 Fig Age at self report in the final sample, broken down by wave. The first 2 waves of self report SWAN scores were acquired from computer assisted telephone interviews between February and December 2009 (wave 1) and between December 2009 and November 2011 (wave 2). The third wave started in August 2012 using an online questionnaire, recruitment is still ongoing. Wave 1 was retrospective (“think back when you were in primary school”) while the 2 following were prospective.
(TIFF)

S4 Fig Self reported SWAN scores for individuals with gross motion during RS-fMRI. Two individuals scored 0 for Inattention and Hyperactivity/Impulsivity, which explains that there are only 3 vertical bars.
(TIFF)

S5 Fig Mother reported SWAN scores of individuals with gross motion. (TIFF)

S6 Fig Boxplot of the head motion distribution per sex and zygosity groups. FMZ: females monozygotic group, MMZ: male monozygotic, FDZ: females from same sex dizygotic pairs, MDZ: males from same sex dizygotic pairs, FOSDZ: females from opposite sex DZ pairs, MOSDZ: males from opposite-sex DZ pairs.
(TIFF)

S1 File Contains Supplementary Appendix A: SWAN samples wave and design description, Table A: Description of the full sample of Self Reported SWAN scores: N = 2,246. Self reported scores were acquired in 3 overlapping waves: N = 373, N = 707 and N = 1,546 (ongoing wave). SWAN questions were retrospective in wave 1 (“think back when you were in primary school”) while they were not in the 2 following (“compare yourself to other people of the same age”). Waves 1 and 2 were acquired over the telephone, wave 3 using an online questionnaire. When several observations were available for one individual we kept the scores acquired in wave 3 (ongoing), than wave 2 and wave 1. This resulted in a total sample of 2,246 individuals. Supplementary Appendix B: Characteristics of individuals with “gross motion”. Table B: Description of the final twin sample and comparison with individuals excluded for gross motion. † We used a Fisher exact test instead of χ2 test to overcome the issue of small sample sizes. We used the Mann–Whitney–Wilcoxon test instead of the t-test because of the greater robustness of its test statistic to individual observations. In addition, our sample contains related family members (within and across groups to compare), thus the variance of heritable traits could be underestimated, leading to a lowering bias on the p-values. To limit the inflation of true positives, we calculated the p-values after excluding related individuals. For the testing, the 2 groups reduced to: N = 8 individuals with gross motion, N = 523 independent included individuals (373 with mother reported score, 446 with self reported). Mean, sd and frequencies reported in the table correspond to the whole sample (includes related individuals). Table C: Results of saturated models for Head Motion, mother and self reported SWAN scores ₫ In self report the design is constant within waves, thus score differences caused by design are captured in the wave effect. This testing ensures that the mean, variances and covariances are homogeneous across sex, birth order and zygosity allowing reducing the model to 2 groups of twins: MZ and DZ. We also tested the effect of age, sex, wave, design and number of siblings by including them in the model. For of self-reported Inattention, the variance difference between females DZ (σ^2^ = 0.39) and MZ (σ^2^ = 0.27, p-value = 0.025) appeared driven by one extreme value (inattention score of 2) in the DZ group (p-value = 0.09 when individual removed). In addition, HM variance was significantly different between females from same sex pairs (σ^2^ = 0.10) and females from opposite sex pairs (σ^2^ = 0.17, p-value = 2.1E-4). Most of the difference was driven by more females from opposite-sex pairs exhibiting high HM, similar to their male co-twin (S6 Fig). The difference could be partially attributed to greater age variability (σ^2^ = 12.5) compared to the female same sex pair groups (σ^2^ = 9.8) but cannot be accounted for by the presence of siblings in the DZ groups (HM variance unchanged after exclusion) nor by a greater resemblance to the male co-twin (similar twin pair correlation as female same-sex group). Finally, we did not model the effect of time difference between scoring and scanning explicitely, but by including both age at questionnaire and age at scan as covariates, we implicitely model the effect of interval time as our model: “SWANscore = b1*AgeQuestionnaire + b2* AgeScan + … + e” is equivalent to: “SWANscore = b1 * (AgeQuestionnaire–AgeScan) + (b1+b2)*AgeScan + … + e”. The Null hypotheses associated with the tests performed are: “Birth order”: Mean/Variance is equal between first and last born twin. “Same sex pairs”: Mean/Variance is equal between female MZ and DZ (same sex) pairs AND Mean/Variance is equal between male MZ and DZ (same sex) pairs. “Same-sex vs opposite-sex pairs”: Mean is equal across all zygosity groups (same sex and opposite sex) for females AND Mean is equal across all zygosity groups (same sex and opposite sex) for males. “Sex groups”: Mean/Variance is equal across sexes. “Quantitative sex-limitation”: Pair covariance equal within same sex zygosity groups. “Qualitative sex limitation”: Pair covariance equal within zygosity groups. “Presence of genetic factors”: Pair covariance equal across zygosity groups. “Familial aggregation”: Pair covariance is null. “Covariate”: Covariate effect is null. Supplementary Appendix C: Heritability of HM and ADHD. Table D: Heritability of SWAN scores and HM. † The models used on the SWAN scores are 2 groups (MZ and DZ) ACE or ADE models for, except for MR Hyperactivity/ Impulsivity where a general sex limitation model was used. As a result, we estimated 7 parameters instead of 3: sex specific A, C/D and E and genetic correlation between sexes. The genetic correlation was exactly 1 in the models leaving the fit unchanged when set to constant. We concluded that the same genetic sources of variance influence mother reported hyperactivity-Impulsivity for males and females, with however differences in intensity. Head motion twin model included age at scan as covariate and allowed the variance to vary between same sex and opposite sex zygosity groups. Best univariate models were AE models for Hyperactivity-Impulsivity (self and mother report), as well as for motion. For the two Inattention scores, ADE models had a significant better fit to the data than AE as the heritability of the trait appears to come mostly from dominant sources of variance (as suggested by large significant D and smaller A).
(DOCX)
