# Supplementary figures and images for: Head Motion and Inattention/Hyperactivity Share Common Genetic Influences: Implications for fMRI Studies of ADHD
Source: PLoS One. 2016 Jan 8;11(1):e0146271. doi: 10.1371/journal.pone.0146271 (PMC4712830; doi:10.1371/journal.pone.0146271)

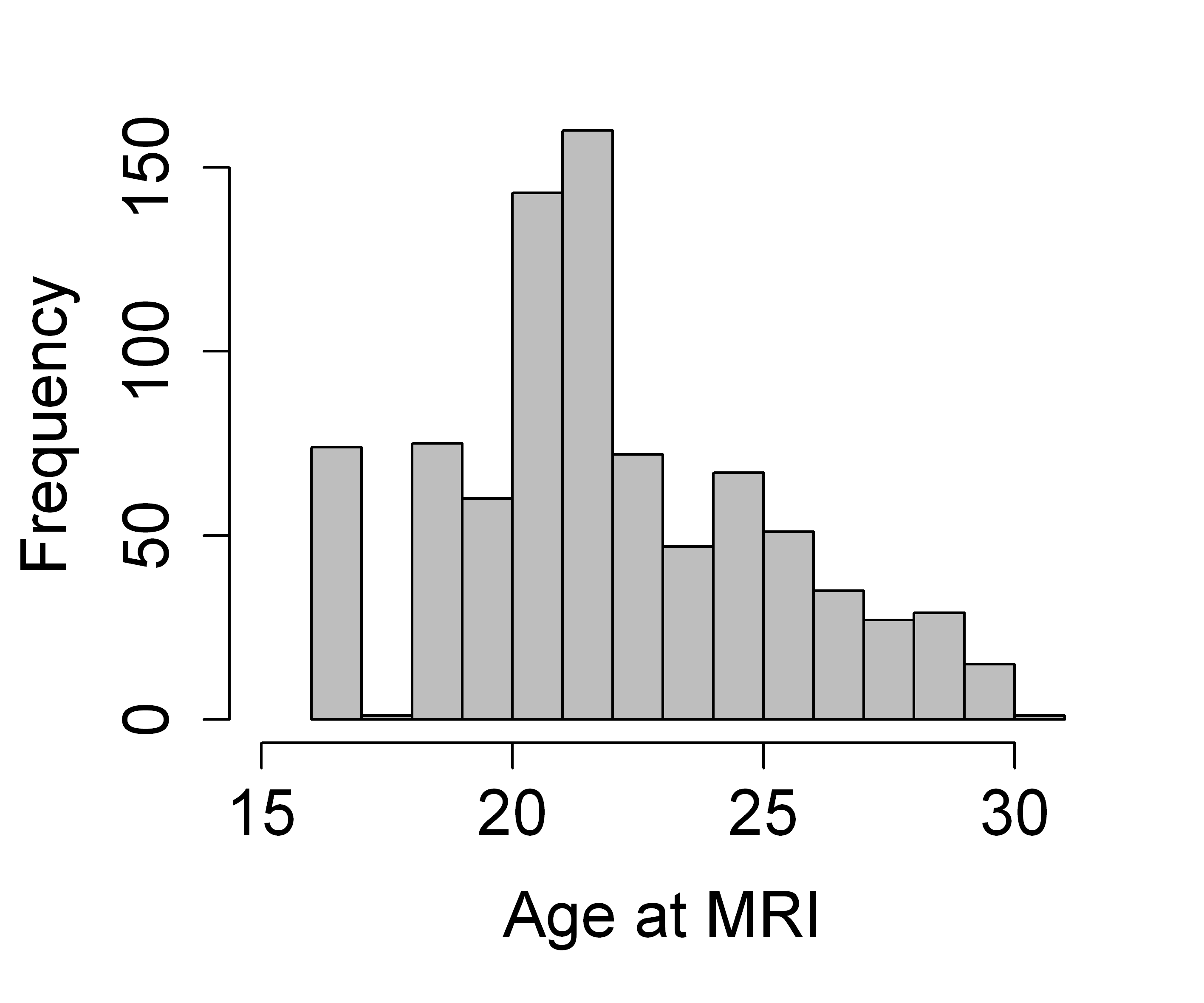

Supplement: S1 Fig — (TIFF) [file pone.0146271.s001.tiff]

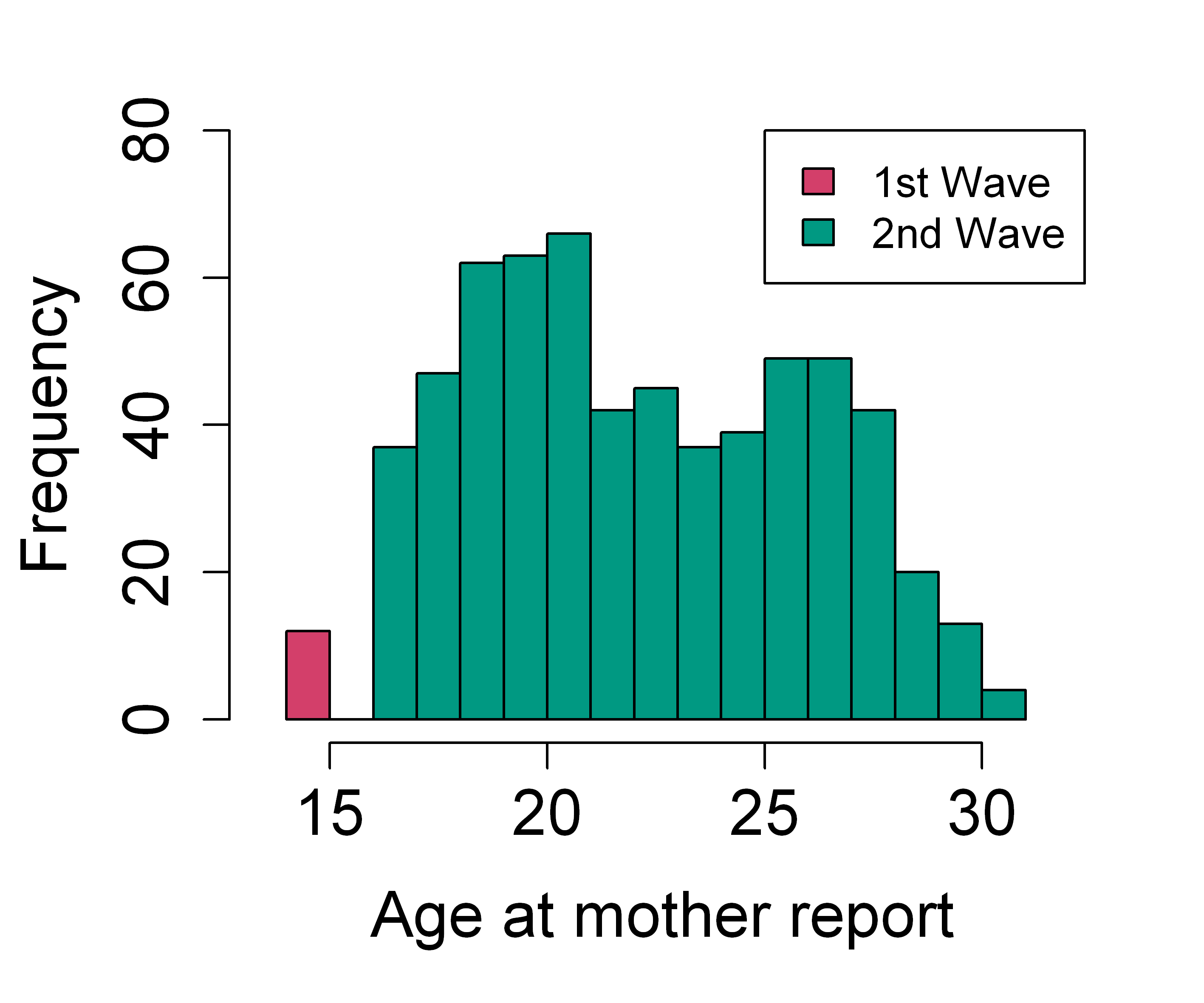

Supplement: S2 Fig — Wave 1 corresponds to mother report acquired during the clinical visits of the twins at age 14. Wave 2 was collected using an online questionnaire several years after the clinical visits. If the twins were older than 20 years old when mothers were contacted, they were asked to rate current symptoms (prospective design). If twins were not 20, mothers were instructed to “think back to when he/she was in primary school” (retrospective design). (TIFF) [file pone.0146271.s002.tiff]

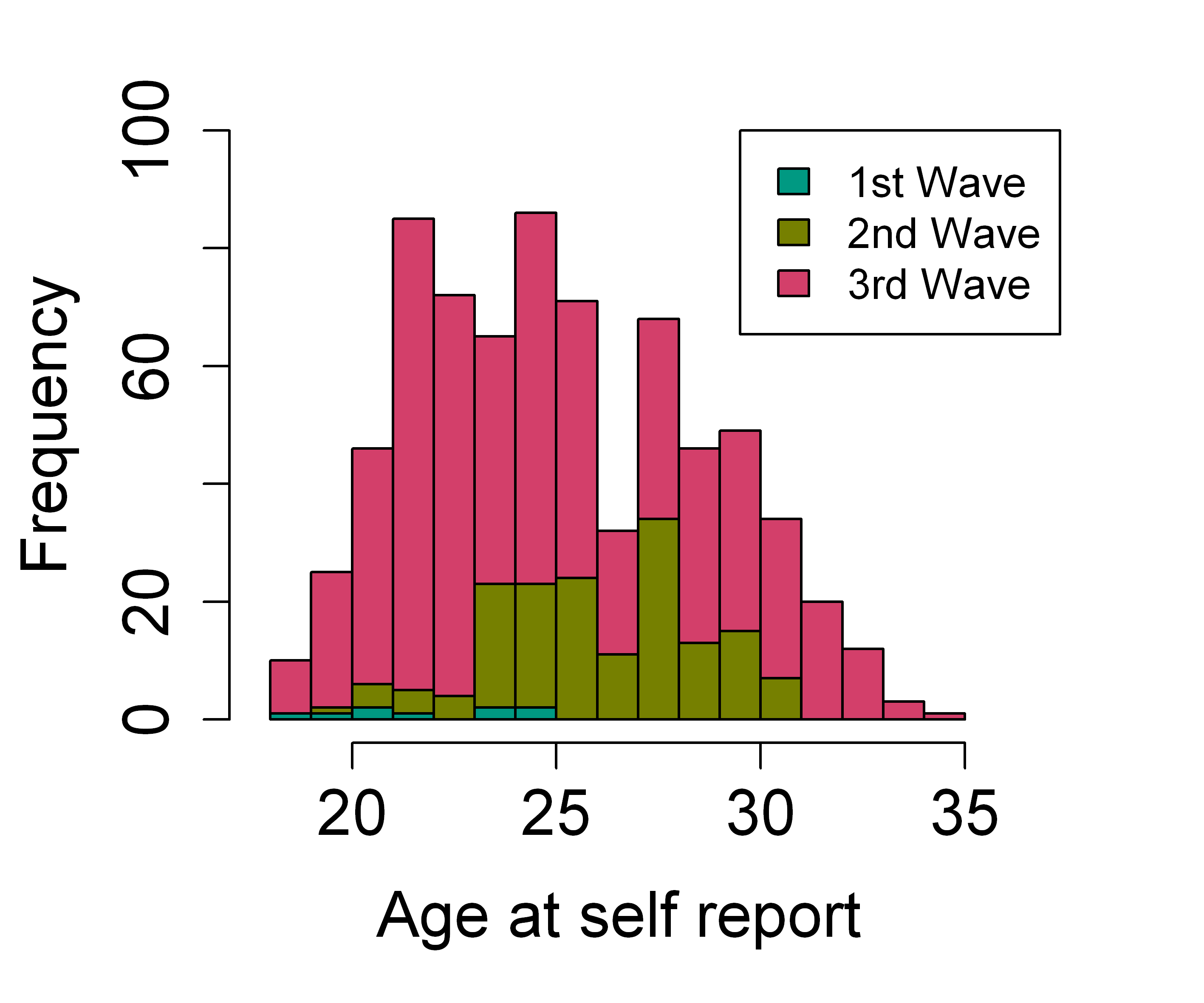

Supplement: S3 Fig — The first 2 waves of self report SWAN scores were acquired from computer assisted telephone interviews between February and December 2009 (wave 1) and between December 2009 and November 2011 (wave 2). The third wave started in August 2012 using an online questionnaire, recruitment is still ongoing. Wave 1 was retrospective (“think back when you were in primary school”) while the 2 following were prospective. (TIFF) [file pone.0146271.s003.tiff]

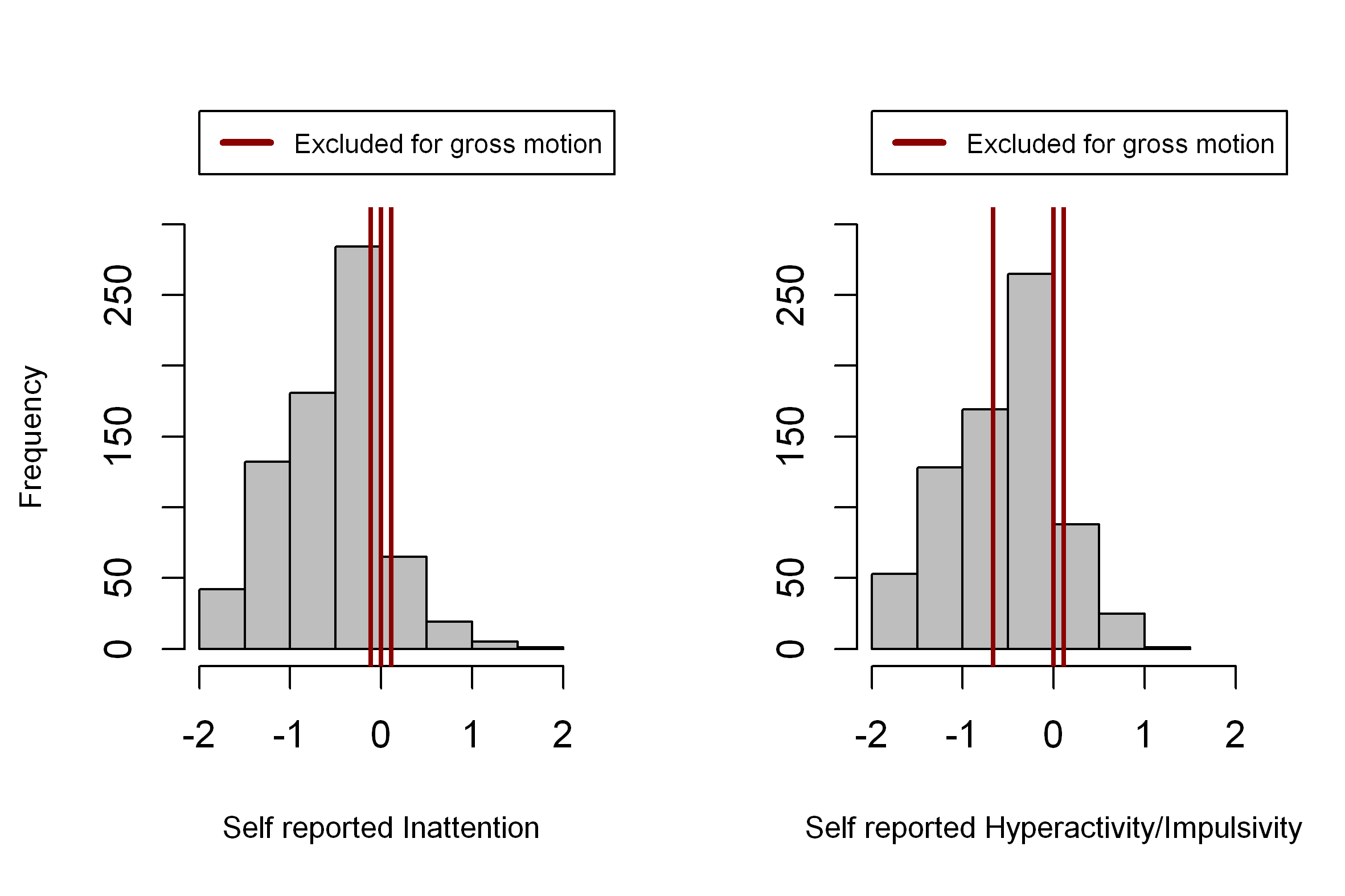

Supplement: S4 Fig — Two individuals scored 0 for Inattention and Hyperactivity/Impulsivity, which explains that there are only 3 vertical bars. (TIFF) [file pone.0146271.s004.tiff]

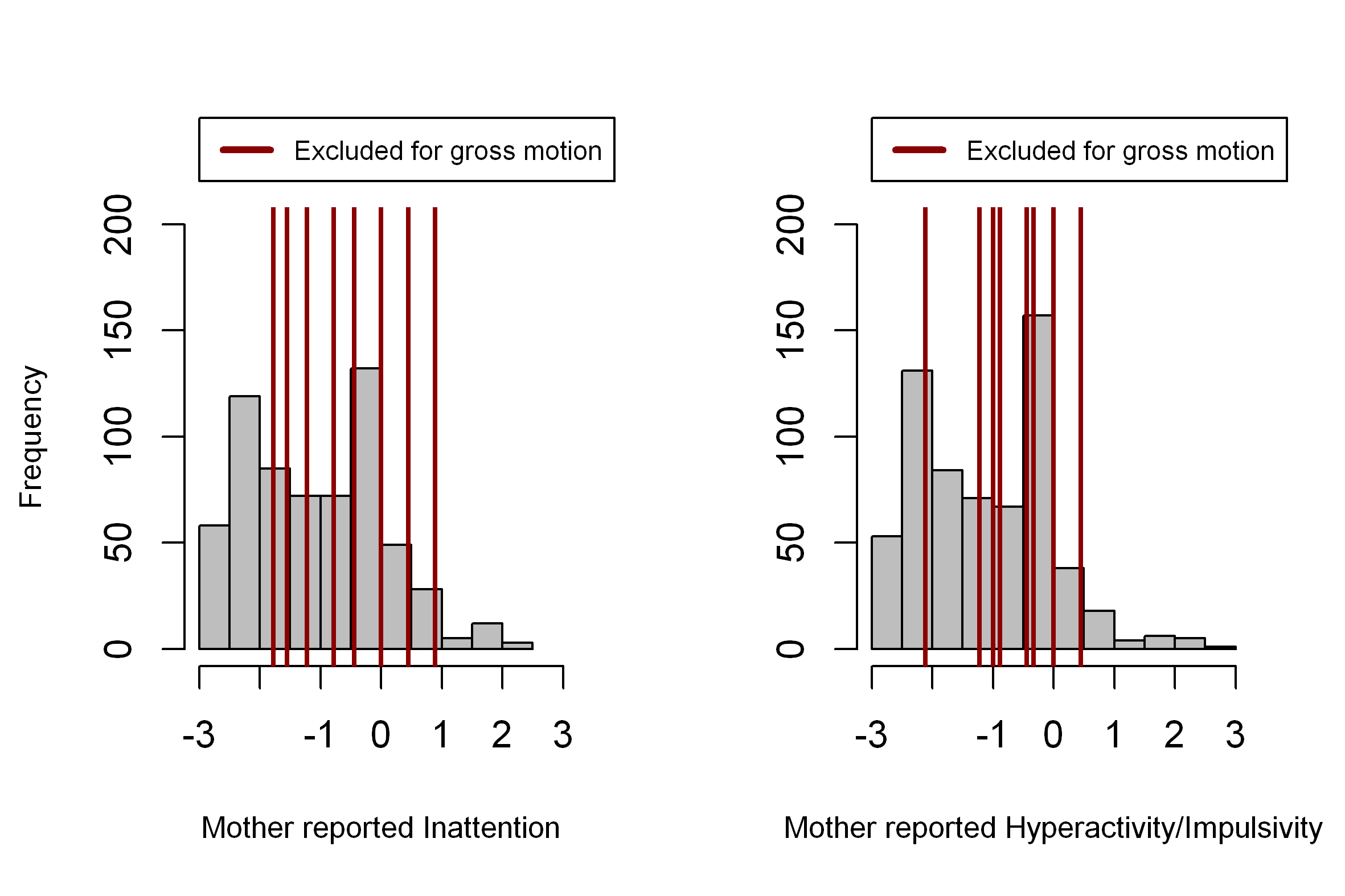

Supplement: S5 Fig — (TIFF) [file pone.0146271.s005.tiff]

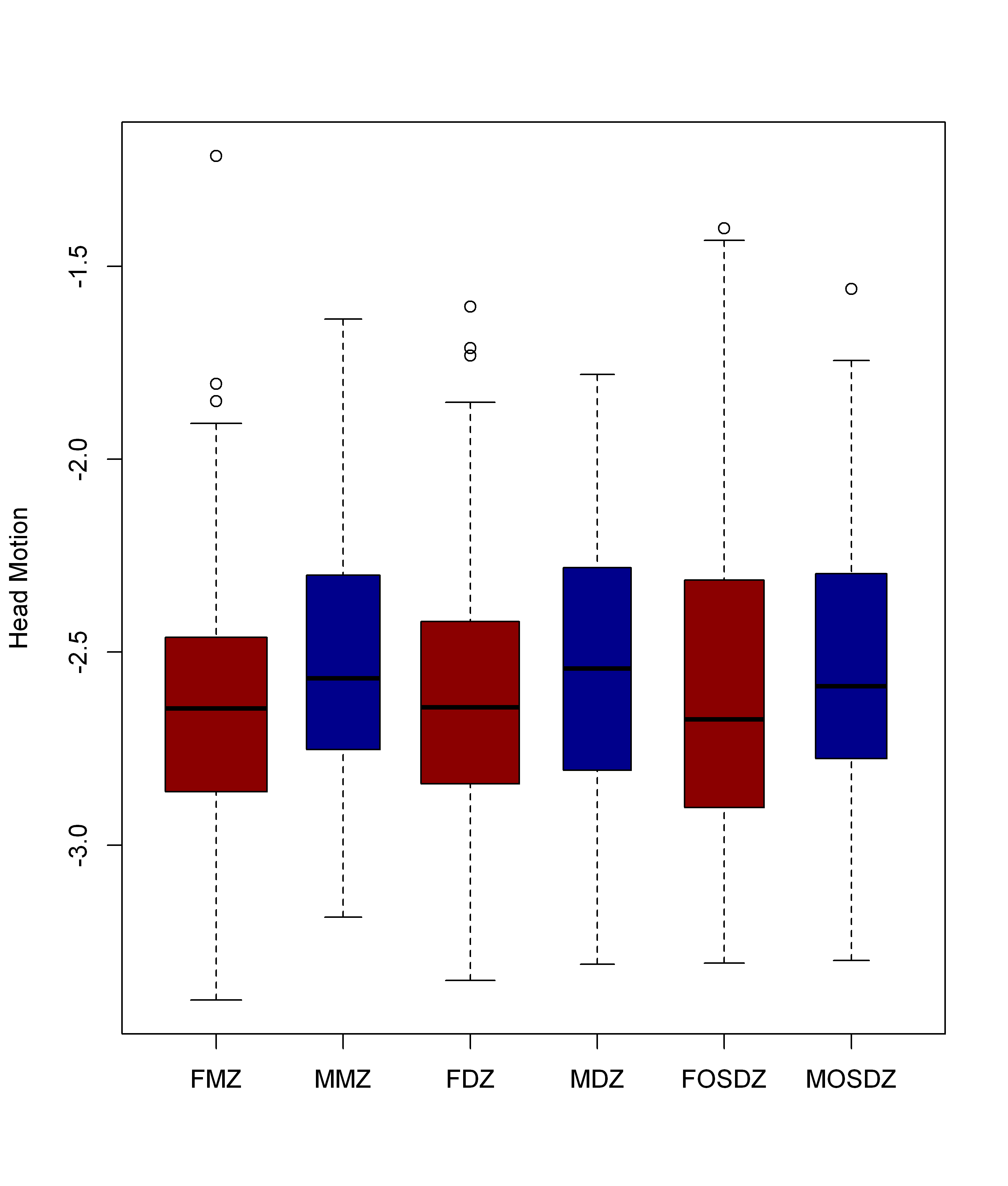

Supplement: S6 Fig — FMZ: females monozygotic group, MMZ: male monozygotic, FDZ: females from same sex dizygotic pairs, MDZ: males from same sex dizygotic pairs, FOSDZ: females from opposite sex DZ pairs, MOSDZ: males from opposite-sex DZ pairs. (TIFF) [file pone.0146271.s006.tiff]
