# Supplementary material for: Head Motion and Inattention/Hyperactivity Share Common Genetic Influences: Implications for fMRI Studies of ADHD
Source: PLoS One. 2016 Jan 8;11(1):e0146271. doi: 10.1371/journal.pone.0146271 (PMC4712830; doi:10.1371/journal.pone.0146271)
Supplement: S1 File — † The models used on the SWAN scores are 2 groups (MZ and DZ) ACE or ADE models for, except for MR Hyperactivity/ Impulsivity where a general sex limitation model was used. As a result, we estimated 7 parameters instead of 3: sex specific A, C/D and E and genetic correlation between sexes. The genetic correlation was exactly 1 in the models leaving the fit unchanged when set to constant. We concluded that the same genetic sources of variance influence mother reported hyperactivity-Impulsivity for males and females, with however differences in intensity. Head motion twin model included age at scan as covariate and allowed the variance to vary between same sex and opposite sex zygosity groups. Best univariate models were AE models for Hyperactivity-Impulsivity (self and mother report), as well as for motion. For the two Inattention scores, ADE models had a significant better fit to the data than AE as the heritability of the trait appears to come mostly from dominant sources of variance (as suggested by large significant D and smaller A). (DOCX) [file pone.0146271.s007.docx]

## Supplementary Appendix A: SWAN samples wave and design description

Twins were on average, 22 years old at mother SWAN report, and 26 years old at self SWAN report, with large age differences within and between waves (S1-3 Fig.). Mother scoring largely (98%) came from an online instrument (wave 2) and only 12 individuals (2%) were assessed when the mother was attending the clinic (at a younger mean twin age of 14: wave1) (Table A in S1 File and Fig. 2). Wave 1 was prospective while for wave 2, 65% of twins were older than 20 at the time of mother report, and were thus scored about their current symptoms (prospective design), with the remaining 35% rated retrospectively (“how was he/she in primary school?”). Self report ratings were collected using an online questionnaire (wave 3: 77%) or telephone interview (wave 2: 22%) at mean age 26 (sd=2.4) and 25 (sd=3.6) respectively, where participants were asked about their current status. The remaining 1% (wave 1) of the self-reported SWAN scores were collected by telephone, at mean age 22 (sd=2.1) and focused on symptoms at primary school (Table A in S1 File and S3 Fig.).

|  | **Wave1** | **Wave2** | **Wave3** | **Total** |
| --- | --- | --- | --- | --- |
| **N (%)** | 38 (2%) | 661 (29%) | 1,546 (69%) | 2,246 |
| **Design** | Retrospective | Prospective | Prospective |  |
| **Acquisition method** | Telephone interview | Telephone interview | Online questionnaire |  |
| **Age (sd)** | 23.6 (3.1) | 27.2 (3.0) | 26.2 (4.2) | 26.4 (3.9) |
| **Females (%)** | 20 (53%) | 380 (57%) | 906 (59%) | 1306 (58%) |
| **Inattention (sd)** | -0.67 (0.58) | -0.46 (0.68) | -0.43 (0.59) | -0.44 (0.62) |
| **Hyperactivity/ Impulsivity (sd)** | -0.53 (0.71) | -0.52 (0.69) | -0.43 (0.62) | -0.46 (0.64) |

Table A: Description of the full sample of Self Reported SWAN scores: N=2,246
Self reported scores were acquired in 3 overlapping waves: N=373, N=707 and N=1,546 (ongoing wave). SWAN questions were retrospective in wave 1 (“think back when you were in primary school”) while they were not in the 2 following (“compare yourself to other people of the same age”). Waves 1 and 2 were acquired over the telephone, wave 3 using an online questionnaire. When several observations were available for one individual we kept the scores acquired in wave 3 (ongoing), than wave 2 and wave 1. This resulted in a total sample of 2,246 individuals

## Supplementary Appendix B: Characteristics of individuals with “gross motion”

Sex and wave frequencies were comparable between individuals included in the final sample and those excluded for gross motion (Table A in S1 File). However, the excluded observations (i.e. 8 individuals) corresponded to significantly younger twins at mother report (mean age 19 vs. 22, p-value=0.015) and RS-fMRI scan (mean age 19 vs. 22, p-value=0.025) (Table A in S1 File). Self reported inattention was significantly lower (p-value=0.028) in the gross motion group, even if only 4 individuals composed this group, which limits the confidence in the test result (S4 Fig.). All other differences did not reach significance. Overall, the individuals excluded for gross motion had higher scores for ADHD (by about 0.5*SD), but larger samples sizes are required to confirm such difference and the significant results reported above (S4 and S5 Figs.). Here, it is unlikely that gross motion exclusion would bias our results.

|  | **Excluded for gross motion (N=8)** | **Included  (N=857)** | **p-value†** | |
| --- | --- | --- | --- | --- |
|  | *Mean (sd) or Frequency (%)* | |  | |
| **Age** (at RS-fMRI) | 19.2 (2.3) | 22.0 (3.2) | **0.015** | |
| **Sex** (female) | 6 (75%) | 545 (64%) | 0.72 | |
| ***Mother reported data*** | *N=8* | *N=627* |  |  |
| **Twin Age** (at questionnaire) | 19.4 (2.2) | 22.1 (3.8) | **0.025** | |
| **Wave**  *“online”* | 8 (100%) | 615 (98%) |  | |
| *“in clinic”* | 0 | 12 (2%) | 1 | |
| **Inattention** | -0.56 (0.96) | -1.04 (1.14) | 0.19 | |
| **Hyperactivity/ Impulsivity** | -0.69 (0.79) | -1.06 (1.10) | 0.38 | |
| ***Self reported data*** | N=4 | N=725 |  | |
| **Twin Age** (at questionnaire) | 23.0 (1.6) | 25.1 (3.4) | 0.15 | |
| **Wave** |  |  |  | |
| *“telephone retrospective”* | 0 | 9 (1%) |  | |
| *“telephone prospective”* | 1 (25%) | 159 (22%) |  | |
| *“online prospective”* | 3 (75%) | 557 (77%) | 0.54 | |
| **Inattention** | 0.00 (0.09) | -0.51 (0.59) | **0.028** | |
| **Hyperactivity/ Impulsivity** | -0.14 (0.36) | -0.50 (0.62) | 0.17 | |

Table B: Description of the final twin sample and comparison with individuals excluded for gross motion

† We used a Fisher exact test instead of χ^2^ test to overcome the issue of small sample sizes. We used the Mann–Whitney–Wilcoxon test instead of the t-test because of the greater robustness of its test statistic to individual observations.
In addition, our sample contains related family members (within and across groups to compare), thus the variance of heritable traits could be underestimated, leading to a lowering bias on the p-values. To limit the inflation of true positives, we calculated the p-values after excluding related individuals. For the testing, the 2 groups reduced to: N=8 individuals with gross motion, N=523 independent included individuals (373 with mother reported score, 446 with self reported). Mean, sd and frequencies reported in the table correspond to the whole sample (includes related individuals).

|  | **Mother Reported Scores** | | | | | | | | **Self Reported Scores** | | | | | | | **Head Motion** | | |
| --- | --- | --- | --- | --- | --- | --- | --- | --- | --- | --- | --- | --- | --- | --- | --- | --- | --- | --- |
|  | **Inattention** | | | | **Hyperactivity / Impulsivity** | | | | **Inattention** | | | | **Hyperactivity / Impulsivity** | | |  |  |  |
|  | *Β (sd)* | | | *p-value* | *Β (sd)* | | | *p-value* | *Β (sd)* | | | *p-value* | *Β (sd)* | | *p-value* | *Β (sd)* | | *p-value* |
| ***Covariates effect and significance*** | | | | | | | | | | | | | | | | | | |
| **Age** *(RS-fMRI)* | -0.051 (0.044) | | | 0.25 | -0.022 (0.046) | | 0.63 | | -0.007 (0.020) | | 0.72 | | 0.006 (0.021) | | 0.77 | **-0.014 (0.004)** | | **4.0E^-4^** |
| **Age** *(at questionnaire)* | 0.047 (0.044) | | | 0.28 | 0.029 (0.046) | | 0.53 | | 0.010 (0.018) | | 0.59 | | 0.0003 (0.019) | | 0.98 |  | | NA |
| **Sex** : *F vs M* | **-0.56 (0.10)** | | | **2.0E^-8^** | **-0.33 (0.09)** | | **2.9E^-4^** | | **-0.18 (0.05)** | | **4.7E^-4^** | | **-0.20 (0.05)** | | **1.5E^-4^** | **-0.081 (0.025)** | | **1.4E^-3^** |
| **Wave:** |  | | |  |  | |  | |  | |  | |  | |  |  | |  |
| **2^nd^ (vs 1st)** | 0.48 (0.40) | | | 0.23 | 0.53 (0.44) | | 0.22 | | 0.19 (0.20) | | 0.34 | | -0.16 (0.21) | | 0.45 |  | | NA |
| **3^rd^ (vs 1st)** |  | | |  |  | |  | | 0.08 (0.19) | | 0.68 | | -0.33 (0.21) | | 0.11 |  | | NA |
| **Design**: *Retrospective vs Prospective* | 0.047 (0.17) | | | 0.78 | 0.20 (0.17) | | 0.25 | |  | | NA₫ | |  | | NA₫ |  | | NA |
| **Number of siblings** | -0.022 (0.050) | | | 0.66 | -0.016 (0.052) | | 0.77 | | 0.015 (0.023) | | 0.50 | | -0.004 (0.002) | | 0.86 |  | | NA |
| **Zygosity Groups Homogeneity**  ***Mean Differences*** | | | | | | | | | | | | | | | | | | |
| “Birth order” |  | | | 0.35 |  | | 0.055 | |  | | | 0.14 |  | | 0.43 |  | | 0.29 |
| “Same sex pairs” |  | | | 0.83 |  | | 0.68 | |  | | | 0.75 |  | | 0.72 |  | | 0.84 |
| “Same-sex vs opposite-sex pairs” |  | | | 0.28 |  | | 0.11 | |  | | | 0.27 |  | | 0.85 |  | | 0.48 |
| “Sex groups” |  | | | 1.0 |  | | 1.0 | |  | | | 1.0 |  | | 1.0 |  | | 1.0 |
| ***Variance Differences*** | | | | | | | | | | | | | | | | | | |
| “Birth order” |  | | | 0.13 |  | | | 0.11 |  | | | 0.87 |  | | 0.49 |  | | 0.93 |
| “Same sex pairs” |  | | | 0.12 |  | | | 0.40 |  | | | **0.024** |  | | 0.11 |  | | 0.76 |
| “Same-sex vs opposite-sex pairs” |  | | | 0.62 |  | | | 0.67 |  | | | 0.71 |  | | 0.81 |  | | **2.1E^-4^** |
| “Sex groups” |  | | | 0.25 |  | | | 0.64 |  | | | 0.33 |  | | 0.18 |  | | 0.72 |
| ***Covariance Difference*** | | | | | | | | | | | | | | | | | | |
| “Quantitative sex-limitation” |  | | | 0.21 |  | **5.8E^-6^** | | |  | 0.24 | | |  | 0.59 | |  | 0.25 | |
| “Qualitative sex-limitation” |  | | | 0.44 |  | NA | | |  | 0.62 | | |  | 0.48 | |  | 0.67 | |
| “Presence of genetic factors” |  | **1.7E^-10^** | | |  | NA | | |  | **3.2E^-5^** | | |  | **0.015** | |  | **2.2E^-4^** | |
| “Familial aggregation” |  | | NA | |  | NA | | |  | NA | | |  | NA | |  | NA | |

Table C: Results of saturated models for Head Motion, mother and self reported SWAN scores

₫ In self report the design is constant within waves, thus score differences caused by design are captured in the wave effect.
This testing ensures that the mean, variances and covariances are homogeneous across sex, birth order and zygosity allowing reducing the model to 2 groups of twins: MZ and DZ. We also tested the effect of age, sex, wave, design and number of siblings by including them in the model. For of self-reported Inattention, the variance difference between females DZ (σ^2^=0.39) and MZ (σ^2^=0.27, p-value=0.025) appeared driven by one extreme value (inattention score of 2) in the DZ group (p-value=0.09 when individual removed). In addition, HM variance was significantly different between females from same sex pairs (σ^2^=0.10) and females from opposite sex pairs (σ^2^=0.17, p-value=2.1E-4). Most of the difference was driven by more females from opposite-sex pairs exhibiting high HM, similar to their male co-twin (S6 Fig.). The difference could be partially attributed to greater age variability (σ^2^=12.5) compared to the female same sex pair groups (σ^2^=9.8) but cannot be accounted for by the presence of siblings in the DZ groups (HM variance unchanged after exclusion) nor by a greater resemblance to the male co-twin (similar twin pair correlation as female same-sex group). ***Finally, we did not model the effect of time difference between scoring and scanning explicitely, but by including both age at questionnaire and age at scan as covariates, we implicitely model the effect of interval time as our model: “SWANscore = b1*AgeQuestionnaire + b2* AgeScan + … + e” is equivalent to: “SWANscore = b1 * (AgeQuestionnaire – AgeScan) + (b1+b2)*AgeScan + … + e”***

The Null hypotheses associated with the tests performed are:
“Birth order”: Mean/Variance is equal between first and last born twin
“Same sex pairs”: Mean/Variance is equal between female MZ and DZ (same sex) pairs AND Mean/Variance is equal between male MZ and DZ (same sex) pairs
“Same-sex vs opposite-sex pairs”: Mean is equal across all zygosity groups (same sex and opposite sex) for females AND Mean is equal across all zygosity groups (same sex and opposite sex) for males
“Sex groups”: Mean/Variance is equal across sexes
“Quantitative sex-limitation”: Pair covariance equal within same sex zygosity groups
“Qualitative sex limitation”: Pair covariance equal within zygosity groups
“Presence of genetic factors”: Pair covariance equal across zygosity groups
“Familial aggregation”: Pair covariance is null
“Covariate”: Covariate effect is null

## Supplementary Appendix C: Heritability of HM and ADHD

We reported summary of univariate models for HM and the SWAN scores in Table D in S1 File. Best models are the simplest (i.e. with the fewest parameters) without significant reduction in fit compared to the full (ACE or ADE) model, they appear in bold (S4). ACE and ADE models are not nested but can be compared using the Akaike Information Criteria (AIC) [[1](#_ENREF_1)].

Heritability of mother reported scores has already been reported for the full sample [[2](#_ENREF_2)]. Here, our final sample comprised 19% of the mother scorings but however yielded identical results. For Inattention, we measured the additive component to be between 0 and 43% (0-35% in [[2](#_ENREF_2)]) and the dominant between 32 and 83% (37-74% in [[2](#_ENREF_2)]). For Hyperactivity-Impulsivity we found the additive sources to explain between 88 and 94% of the variance in females (79-94% in [[2](#_ENREF_2)]). The A component was only explaining 63 to 87% in males (72-85% in [[2](#_ENREF_2)]). The general sex limitation model outperformed standard ACE fit (p-value=5.1E-6), confirming tests from the hypothesis testing (Table C in S1 File). Genetic correlation (r_g_=1) between males and females suggested that genes influencing Hyperactivity-Impulsivity are the same, even if they explain different proportion of variance within sexes. Similar heritability estimates have also been reported in an independent sample using the SWAN instrument [[3](#_ENREF_3)].

In heritability of self reported scores, our sample only overlaps partially with Ebejer et al., [[2](#_ENREF_2)] due to ongoing recruitment (see Material and Methods). In addition they modeled the negative contrast effects by including siblings of twin pairs in the analysis [[2](#_ENREF_2)], who were not imaged. Such contrast often results in underestimation of MZ and DZ correlations, which may spuriously suggest (or bias upward) dominance estimates. In addition it can lead to an overestimation of variance [[4](#_ENREF_4), [5](#_ENREF_5)] that reduces power and can even bias heritability estimates (when more pronounced in MZ or DZ group). Controlling the scores for the number of siblings as previously suggested [[6](#_ENREF_6)] had no effect on the rater’s effect (Table A in S1 File). Therefore, we could expect our results to show wider confidence intervals (power reduction caused by negative contrast and smaller sample size) with larger dominant sources of variance than Ebejer’s [[2](#_ENREF_2)]. However, we observed very comparable results for both Inattention and Hyperactivity-Impulsivity: small estimates of additive genetic sources of variance with large CI (that include 0), medium estimates of dominant sources of variance with again large CI, though largely overlapping across studies (Table D in S1 File) [[2](#_ENREF_2)]. Both studies lack power to detect small additive genetics and dominant effect, which is caused by limited sample size (here) or report of estimates for males and females [[2](#_ENREF_2)] which eliminated the increased precision in estimation that a larger study had. The agreement of results (largely overlapping CIs) was also enhanced by the contrast effect being small [[2](#_ENREF_2)]. Heritability reported in an independent sample also found self-report to be less heritable (of about 20%). This difference might be greater in our sample, even if the absence of distinction between heritability of self and parental report [[3](#_ENREF_3)] makes a detailed comparison impossible.

Finally, heritability of HM was consistent with estimates previously calculated on a subset of 462 twins [[7](#_ENREF_7)]. The addition of 67 extra twin pairs led to slightly more precise estimate: h^2^_HM_ =0.40 [0.26,0.53] (against h^2^_HM_=0.49 [0.32,0.62] previously).

| **Variable** | **Model**† | | **Parameter estimates** | | | | |  | | |  | | **Model fit** | | | |
| --- | --- | --- | --- | --- | --- | --- | --- | --- | --- | --- | --- | --- | --- | --- | --- | --- |
|  | |  | | **A** | **C/D** | **E** | **df** | | **Δdf** | | | **-2LL** | | **Δ-2LL** | **AIC** | **P-value** |
| **Mother reported Inattention** | | **ADE** | | **0.00 [0.00,0.43]** | **0.77 [0.32,0.83]** | **0.23 [0.17,0.32]** | | **621** | |  | | **1837.6** | |  | **595.6** |  |
|  |  | AE | | 0.73 [0.62,0.81] |  | 0.27 [0.19,0.38] | | 622 | | 1 | | 1847.7 | | 10.2 | 635.8 | 1.4E-3 |
|  |  | E | |  |  |  | | 623 | | 2 | | 1909.5 | | 71.9 | 695.4 | 2.4E-16 |
|  | | ACE | | 0.73 [0.61,0.81] | 0.00 [0.00,0.07] | 0.27 [0.19,0.38] | | 621 | |  | | 1847.7 | |  | 605.7 |  |
| **Mother reported Hyperactivity Impulsivity** | | ACE f | | 0.71 [0.46,0.94] | 0.21 [0.00,0.46] | 0.08 [0.06,0.12] | |  | |  | |  | |  |  |  |
|  |  | m | | 0.72 [0.32,0.86] | 0.05 [0.00,0.37] | 0.23 [0.13,0.43] | | 619 | |  | | 1686.1 | |  | 448.1 |  |
|  |  | ACE | | 0.80 [0.58,0.90] | 0.07 [0.00,0.28] | 0.13 [0.10,0.18] | | 622 | | 3 | | 1713.4 | | 27.3 | 469.4 | 5.1E-6 |
|  |  | **AE f** | | **0.92 [0.88,0.94]** |  | **0.08 [0.06,0.12]** | |  | |  | |  | |  |  |  |
|  |  | **m** | | **0.79 [0.63,0.87]** |  | **0.21 [0.13,0.37]** | | **621** | | **2** | | **1687.9** | | **1.75** | **445.9** | **0.42** |
|  |  | CE f | |  | 0.74 [0.65,0.80] | 0.26 [0.20,0.35] | |  | |  | |  | |  |  |  |
|  |  | m | |  | 0.41 [0.22,0.58] | 0.59 [0.42,0.78] | | 621 | | 2 | | 1737.5 | | 51.4 | 495.5 | 6.8E-12 |
|  |  | E | |  |  |  | | 623 | | 4 | | 1870.1 | | 184.0 | 624.1 | 1.0E-38 |
|  | | ADE f | | 0.92 [0.58,0.94] | 0.00 [0.00,0.34] | 0.08 [0.06,0.12] | |  | |  | |  | |  |  |  |
|  | | m | | 0.77 [0.21,0.87] | 0.00 [0.00,0.58] | 0.23 [0.13,0.40] | | 619 | |  | | 1687.9 | |  | 449.9 |  |
| **Self reported Inattention** | | **ADE** | | **0.00 [0.00,0.22]** | **0.45 [0.16,0.60]** | **0.55[0.40,0.75]** | | 719 | |  | | **1274.0** | |  | **-164.0** |  |
|  |  | AE | | 0.31 [0.11,0.48] |  | 0.69 [0.52,0.89] | | 720 | | 1 | | 1280.8 | | 6.8 | -159.2 | 9.3E-3 |
|  |  | E | |  |  |  | | 721 | | 2 | | 1290.2 | | 16.2 | -151.8 | 3.0E-4 |
|  |  | ACE | | 0.31 [0.10,0.48] | 0.00 [0.00,0.095] | 0.69 [0.52,0.89] | | 719 | |  | | 1280.8 | |  | -157.2 |  |
| **Self reported Hyperactivity Impulsivity** | | ADE | | 0.00 [0.00,0.27] | 0.28 [0.00,0.43] | 0.74 [0.57,0.95] | | 719 | |  | | 1345.5 | |  | -92.5 |  |
|  |  | **AE** | | **0.17 [0.00,0.34]** |  | **0.83 [0.66,1.00]** | | **720** | | **1** | | **1347.9** | | **2.4** | **-92.1** | **0.12** |
|  |  | E | |  |  |  | | 721 | | 2 | | 1351.5 | | 6.0 | -90.5 | 0.050 |
|  |  | ACE | | 0.17 [0.00,0.34] | 0.00 [0.00,0.14] | 0.83 [0.66,1.00] | | 719 | |  | | 1347.9 | |  | -90.1 |  |
| **Head Motion** | | ADE | | 0.07[0.00,0.50] | 0.38 [0.00,0.57] | 0.55 [0.43,0.71] | | 850 | |  | | 566.7 | |  | -1133.3 |  |
|  |  | **AE** | | **0.40 [0.26,0.53]** |  | **0.60 [0.47,0.74]** | | **851** | | **1** | | **568.3** | | **1.60** | **-1133.7** | **0.21** |
|  |  | E | |  |  |  | | 852 | | 2 | | 593.8 | | 27.12 | -1110.2 | 8.0E-7 |
|  | | ACE | | 0.40 [0.16,0.53] | 0.00 [0.00,0.16] | 0.60 [0.47,0.74] | | 850 | |  | | 568.3 | |  | -1124.7 |  |

Table D: Heritability of SWAN scores and HM

† The models used on the SWAN scores are 2 groups (MZ and DZ) ACE or ADE models for, except for MR Hyperactivity/ Impulsivity where a general sex limitation model was used. As a result, we estimated 7 parameters instead of 3: sex specific A, C/D and E and genetic correlation between sexes. The genetic correlation was exactly 1 in the models leaving the fit unchanged when set to constant. We concluded that the same genetic sources of variance influence mother reported hyperactivity-Impulsivity for males and females, with however differences in intensity.
Head motion twin model included age at scan as covariate and allowed the variance to vary between same sex and opposite sex zygosity groups.
Best univariate models were AE models for Hyperactivity-Impulsivity (self and mother report), as well as for motion. For the two Inattention scores, ADE models had a significant better fit to the data than AE as the heritability of the trait appears to come mostly from dominant sources of variance (as suggested by large significant D and smaller A)

1. Akaike H. A new look at the statistical model identification. Automatic Control, IEEE Transactions on. 1974;19(6):716-23.

2. Ebejer JL, Medland SE, van der Werf J, M JW, Henders AK, Gillespie NA, et al. Contrast Effects and Sex Influence Maternal and Self-Report Dimensional Measures of Attention-Deficit Hyperactivity Disorder. Behav Genet. 2014. doi: 10.1007/s10519-014-9670-x. PubMed PMID: 25151025.

3. Chang Z, Lichtenstein P, Asherson PJ, Larsson H. Developmental twin study of attention problems: high heritabilities throughout development. JAMA Psychiatry. 2013;70(3):311-8. doi: 10.1001/jamapsychiatry.2013.287. PubMed PMID: 23303526.

4. Simonoff E, Pickles A, Hervas A, Silberg JL, Rutter M, Eaves L. Genetic influences on childhood hyperactivity: contrast effects imply parental rating bias, not sibling interaction. Psychol Med. 1998;28(4):825-37. PubMed PMID: 9723139.

5. Neale MC, Cardon LR. Methodology for genetic studies of twins and families: Kluwer Academic Pub; 1992.

6. Pinto R, Rijsdijk F, Frazier-Wood AC, Asherson P, Kuntsi J. Bigger families fare better: a novel method to estimate rater contrast effects in parental ratings on ADHD symptoms. Behav Genet. 2012;42(6):875-85. doi: 10.1007/s10519-012-9561-y. PubMed PMID: 23053732; PubMed Central PMCID: PMC4189093.

7. Couvy-Duchesne B, Blokland GA, Hickie IB, Thompson PM, Martin NG, de Zubicaray GI, et al. Heritability of head motion during resting state functional MRI in 462 healthy twins. NeuroImage. 2014. doi: 10.1016/j.neuroimage.2014.08.010. PubMed PMID: 25132021.
